# The Treatment of Fractures from a Common-Sense Point of View

**Published:** 1907-11-02

**Authors:** 


### Points in Surgery.

### THE TREATMENT OF FRACTURES FROM A COMMON-SENSE POINT OF VIEW.

### XI. FRACTURES OF THE NECK OF THE FEMUR.

These fractures are divided into intracapsular and extracapsular, according to whether the line of fracture lies inside or outside the capsule of the joint. The capsular ligament is attached to the femur along the anterior inter-trochanteric line in front, and one finger's breadth from the posterior inter-trochanteric line behind. Some of the deeper fibres are reflected upwards along the neck of the femur, and are attached to it nearer the head; they are known as retinacular fibres, and said to play a more or less important part in intracapsular fractures of the neck in preventing excessive separation of the fragments. It is difficult to believe that they really have any action of this kind, since their structure is somewhat frail. The distinction between these two varieties of fracture really rests more on clinical than on anatomical grounds; that is, in each case the nature of the injury and the cause producing it is quite different.

#### Intracapsular Fracture.

Intracapsular fracture occurs in enfeebled elderly people, whose bones are brittle as the result of calcareous absorption, and thus readily broken. The exciting cause is often ridiculously slight; thus catching the foot on an uneven piece of carpet not uncommonly produces it, although the patient does not necessarily fall to the ground until after the bone is broken. The line of fracture is usually quite close to the head of the bone, since this is the portion from which the calcareous salts are earliest absorbed. The following are the clinical signs on which the injury may be diagnosed:—The limb is shortened from half to one inch; it should always be measured if an injury to the bone is suspected. In measuring a limb fixed points should be taken. In the leg it is convenient to measure from the anterior superior spine to the tip of the internal malleolus, the tape being held down at the inner side of the knee at a point equidistant from the anterior and posterior surfaces. Moreover, as measurements are only made in order to compare the sound limb with the injured one, both legs must be held in an identical position while the measurements are being made, or the result will lead one into error. It is obvious, for instance, that if one leg is measured in the extended position and the other in the flexed, the length will vary in each case, although both legs might be sound. The limb is everted; this is due to the natural tendency of the leg, which is now unopposed, to assume an everted position. The patient is unable to move it, but if this be done by the surgeon much pain is evinced. Crepitus is not often obtained. If the limb is passively rotated it will be found that the arc of rotation of the great trochanter is diminished as compared with that on the sound side. The upper surface of the trochanter is pulled above Nélaton's line, which is a line drawn from the anterior superior spine to the tuber ischii. The iliotibial band is not tense, as in a normal limb, but is appreciably slackened. In the presence of so many clinical signs the diagnosis of this injury should not be difficult, but it is none the less a fact that it is sometimes mistaken for osteoarthritis of the hip joint, chiefly because the violence producing the fracture is so slight that its presence is not suspected. In osteoarthritis there may be pain on moving the limb, and a grating sensation that may conceivably be mistaken for crepitus. But in osteoarthritis the onset of pain is gradual, the surface of the trochanter is thickened, and its upper surface is not pulled up above Nélaton's line; and the other characteristic features described above are absent.

In these cases it is almost impossible to obtain sound union on account of the age of the patient, and it is safe to prophesy that the limb is never likely to be a really useful one again. It is more important to treat the patient than the fracture, the main object being to avoid the occurrence of hypostatic pneumonia, a complication most liable to occur in enfeebled old people if they are kept on their backs. The patient should be put to bed, therefore, with the leg between sandbags only for a few days until the pain and swelling have disappeared. Massage is very useful in hastening this. As soon as ever it is possible he should be encouraged to get about on a Thomas' hip splint. The splint should be changed for crutches after six or seven weeks. It is more than likely that the patient will have to continue to use the crutches for the rest of his life, since, even in a favourable case, fibrous union results, and the limb is unable to stand any weight being put upon it.

#### Extracapsular Fracture.

Extracapsular fracture is a very different matter. It differs from the injury just described in almost every particular. It is the result of extreme direct violence, such as a kick from a horse on the trochanter or a fall from a height. The limb is everted, as in the former case, but is shortened as much as two or three inches. The arc of rotation of the trochanter is absent, unless the fracture is impacted; this is not uncommon on account of the great violence producing the fracture. What usually happens is that the neck of the femur is driven into the great trochanter, which splits to receive it. The question arises, Should the bones be disimpacted? It is sometimes better to let the fragments unite as they are, but the following conditions demand disimpaction:—(1) Shortening of more than one inch; (2) malposition of the foot; (3) pressure on important structures, such as the femoral vessels or sciatic nerve.

The only practicable form of treatment for this injury is to apply extension to the limb by means of a weight and pulley and a Liston's splint. The method of applying this was described in a previous article. There are two small points which make a great difference to the patient's comfort. One is that the mattress should be unyielding: to this end, the patient's buttocks should be supported on boards which rest transversely on the framework of the bed, and the other is that the limb should be kept very slightly abducted during the treatment. The weight to be applied can be readily calculated by allowing half a pound for each year of the patient's age up to ten pounds. It is hardly ever necessary to apply more than this. After four or five weeks the extension will have done its work, and the limb can be fixed in a silicate or plaster bandage. The former is the more convenient, because it is lighter and less bulky. The patient should not be allowed to attempt to walk until the tenth week.

